# A maximum flow-based network approach for identification of stable noncoding biomarkers associated with the multigenic neurological condition, autism

**DOI:** 10.1186/s13040-021-00262-x

**Published:** 2021-05-03

**Authors:** Maya Varma, Kelley M. Paskov, Brianna S. Chrisman, Min Woo Sun, Jae-Yoon Jung, Nate T. Stockham, Peter Y. Washington, Dennis P. Wall

**Affiliations:** 1 Department of Computer Science, Stanford University, Stanford, CA USA; 2 Department of Biomedical Data Science, Stanford University, Stanford, CA USA; 3 Department of Bioengineering, Stanford University, Stanford, CA USA; 4 Department of Pediatrics, Stanford University, Stanford, CA USA; 5 Department of Neuroscience, Stanford University, Stanford, CA USA

**Keywords:** Maximum flow, Network, Feature selection, Feature stability, Linkage disequilibrium, Machine learning

## Abstract

**Background:**

Machine learning approaches for predicting disease risk from high-dimensional whole genome sequence (WGS) data often result in unstable models that can be difficult to interpret, limiting the identification of putative sets of biomarkers. Here, we design and validate a graph-based methodology based on maximum flow, which leverages the presence of linkage disequilibrium (LD) to identify stable sets of variants associated with complex multigenic disorders.

**Results:**

We apply our method to a previously published logistic regression model trained to identify variants in simple repeat sequences associated with autism spectrum disorder (ASD); this *L*_1_-regularized model exhibits high predictive accuracy yet demonstrates great variability in the features selected from over 230,000 possible variants. In order to improve model stability, we extract the variants assigned non-zero weights in each of 5 cross-validation folds and then assemble the five sets of features into a flow network subject to LD constraints. The maximum flow formulation allowed us to identify 55 variants, which we show to be more stable than the features identified by the original classifier.

**Conclusion:**

Our method allows for the creation of machine learning models that can identify predictive variants. Our results help pave the way towards biomarker-based diagnosis methods for complex genetic disorders.

**Supplementary Information:**

The online version contains supplementary material available at 10.1186/s13040-021-00262-x.

## Background

The advent of inexpensive whole genome sequencing methods in recent years has led to the creation of supervised machine learning approaches for predicting putative genetic variants from sequence data. Machine learning methods can effectively investigate the entire genome and provide insight into the subset of variants most likely to influence a particular phenotype, which is particularly useful for disorders or traits with complex, non-Mendelian inheritance patterns [1–5]. Since the high dimensionality of variant feature sets paired with a comparatively low number of training samples tends to result in model overfitting, feature selection methods are often used to narrow the genomic search space and improve model generalizability. Embedded selection methods, such as *L*_1_ and *L*_2_ regularization, are a subclass of feature selection algorithms that learn the optimal subset of genomic features during model training.

Although such methods are widely used, regularized machine learning models often face issues related to stability and robustness of features. Specifically, classification models, which are designed to distinguish between case and control populations based on whole genome data, provide coefficient scores representing the contribution of each feature to the classification result. However, slight perturbations to the dataset or model often drastically alter the subset of top-ranked variants determined to be correlated with the phenotype, a phenomenon known as feature instability [2, 6, 7]. The absence of stability among predictive features means that variants with high coefficient scores may not necessarily provide insight into the biological mechanisms underlying a condition. This has particularly been observed in polygenic risk scores, which can tend to vary based on the methods and data used to perform calculations [8]. Feature instability often leads to irreproducible and inconsistent results, making this issue one of the largest hindrances to clinical applications of both biomarkers identified by machine learning techniques and polygenic risk scores [9].

In this work, we present an approach to improve the stability of regularized machine learning methods through incorporation of biological information. We hypothesize that the observed instability of regularized machine learning models trained on large genome datasets results from linkage disequilibrium (LD) among variants. Prior research has shown that in such situations with correlated features, machine learning models can pick from a number of equally predictive features, which leads to unstable results [10, 11]. In addition, the presence of linkage between variants has been previously shown to limit the predictive power of polygenic risk scores as well as cause models to assign high coefficient scores to noncausal variants [12, 13].

To the best of our knowledge, our hypothesis has not been previously explored. Standard methods for handling correlated genomic features include LD pruning, which discards some correlated variants prior to model training, and loss function modification, which assigns an additional regularization penalty based on feature correlation [12, 14, 15]. Prior work has shown that the incorporation of LD information into regularized models through such techniques can improve prediction performance. However, the relationship between LD-based feature selection approaches and model stability has not been extensively studied.

In order to identify a stable set of putative variants resistant to data perturbations, we design an algorithm based on maximum flow, which utilizes the presence of LD to perform biologically-informed feature selection. We demonstrate the efficacy of our algorithm using a previously trained model predicting autism spectrum disorder (ASD), a prevalent neurodevelopmental disorder affecting one in 40 children in the United States [16]. Characterized by social impairments, restricted and repetitive behaviors, and speech and language delay, ASD is known to be approximately 80% heritable, with over 1000 genes contributing to disease susceptibility [17, 18]. In a previous study, we utilized regularized machine learning to show that variants in a specific subclass of noncoding DNA known as simple repeat sequences (SRS) may be predictive of the ASD phenotype [19]. Here, we extend this work by narrowing the search space and identifying a stable, robust set of putative SRS variants potentially linked with ASD. We further validate our proposed algorithm through simulations on synthetic data.

In summary, our contribution in this work is two-fold: (1) we introduce a novel methodology that utilizes LD relationships to improve the stability of regularized machine learning models, and (2) we utilize our method to identify a stable set of SRS variants potentially linked with ASD.

## Results

### Stability analysis

We performed initial measurements of feature stability prior to implementing the maximum-flow formulation. We began by encoding the 232,193 SRS variants into a binary feature matrix, and we performed five-fold cross-validation with an *L*_1_-regularized logistic regression classifier. The hyperparameter *λ* was set to 10 after tuning the model through a grid search; the high regularization penalty allows for the selection of a core set of variants contributing to the classifier output. Further details on hyperparameter selection are included in our prior work [19]. We extracted variants with non-zero coefficient scores from each of the five validation folds, resulting in a mean of 435.6 variants per fold.

**Fig. 1:**
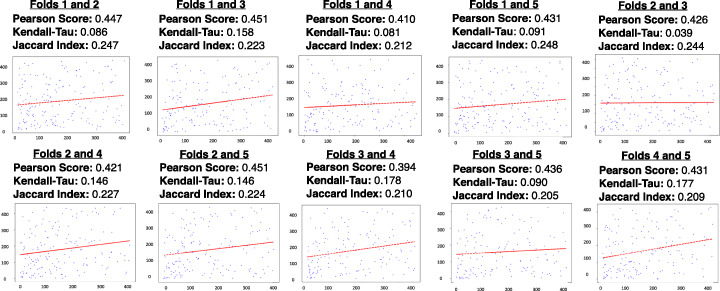
*Initial feature stability metrics*. Pearson correlation coefficients, Kendall-Tau scores, Jaccard similarity indices, and rank plots were calculated for each pairwise grouping of validation folds. This provides a quantitative analysis of feature stability by evaluating the effect of training set perturbations on the resulting top-ranked variant list. The rank plots compare the magnitude of coefficient scores assigned to features across a pair of folds, with the axes representing the relative rank of a particular variant. In the presence of perfect model stability, the ranked variant lists would be equivalent between a pair of folds, resulting in a trend line with a slope of 1. However, in this cross-fold analysis, the scatter plots show a high degree of randomness, with trend line slopes approaching zero

We then performed a pairwise feature stability comparison across all ten pairs of feature lists. We observed Pearson correlation coefficients ranging from 0.394 to 0.451, suggesting a moderate degree of similarity between coefficient scores assigned to a specific feature across folds. Kendall-Tau scores ranged between 0.039 and 0.178, and Jaccard indices ranged between 0.205 and 0.248; this shows that predictive variants identified by the classifier vary drastically as the underlying data is modified (Fig. 1).

We then implemented the maximum flow formulation and constructed a flow network consisting of the five sets of variants with non-zero coefficients, as determined by the 5-fold cross-validation analysis. The network includes 4358 nodes representing variants and 3757 directed edges; 699 of these edges connect a pair of variants in LD across folds, with the remainder connecting in and out nodes within a single fold or connecting nodes to the source and sink. A Ford-Fulkerson search through the graph resulted in a flow value of 51. Each path consists of between one and five unique variants; the 51 paths include 55 distinct variants (Additional file 1).

In order to determine if these results suggest a higher degree of stability than expected by chance, we performed a bootstrap test. We randomly selected five sets of SRS variants and reconstructed the flow network. Results show that all 100 iterations of this test resulted in a maximum flow value of 0. Flow networks had a mean of only 3.99 edges across folds; this resulted in disjointed bootstrap graphs, showing that selecting features at random results in a complete lack of feature stability and that variants in LD are unlikely to be selected in neighboring folds by random chance alone. Thus, our observed maximum flow of 51 supports our hypothesis that the classifier extracts variants in the same genomic regions across different folds, resulting in a likely false perception of instability.

**Fig. 2:**
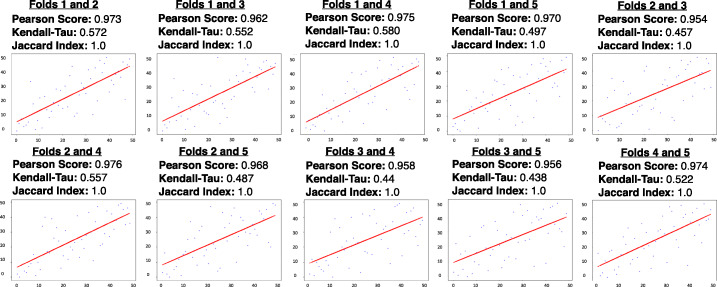
*Feature stability metrics after maximum flow procedure.* After implementing the maximum flow formulation, Pearson correlation coefficients, Kendall-Tau scores, Jaccard similarity indices, and rank plots were recalculated for each pairwise grouping of validation folds, computed with binary representations of the reduced feature set. This demonstrates an increased degree of stability when compared to the initial results in Fig. 1

Finally, we validated the stability of the 55 identified SRS variants by evaluating performance of a logistic regression classifier trained on this reduced feature set. We performed 5-fold cross-validation across the training set and determined the stability of these variants by recomputing the Pearson correlation coefficients, Kendall-Tau scores, and Jaccard similarity indices. Results show a higher degree of stability, with Pearson correlation coefficients ranging between 0.954 and 0.976 and Kendall-Tau scores ranging between 0.438 and 0.580; this suggests that the classifier is assigning similar coefficient scores despite slight perturbations to the underlying data. Since we constrained the feature set to be the same variant groups in each fold, the Jaccard index is trivially 1.0 (Fig. 2). When evaluated on a held-out test set consisting of 425 ASD patients and 76 control individuals, this classifier demonstrated high predictive performance, achieving a precision of 0.914, a recall of 0.821, and an AUC-ROC of 0.810.

### Simulated dataset analysis

In order to further evaluate the maximum flow approach, we conducted additional experiments on simulated data. We generated synthetic feature matrices and evaluated the performance and stability of the following three regularized machine learning models: logistic regression with L_1_ regularization, logistic regression with elastic net regularization, and linear support vector classification (SVC) with L_1_ regularization. High levels of regularization were used in order to encourage maximal dimensionality reduction, with *λ =* 10 for L_1_ regularization, *λ =* 100 for feature correlation experiments with elastic net regularization, and *λ =* 1000 for dataset size experiments with elastic net regularization.

**Fig. 3:**
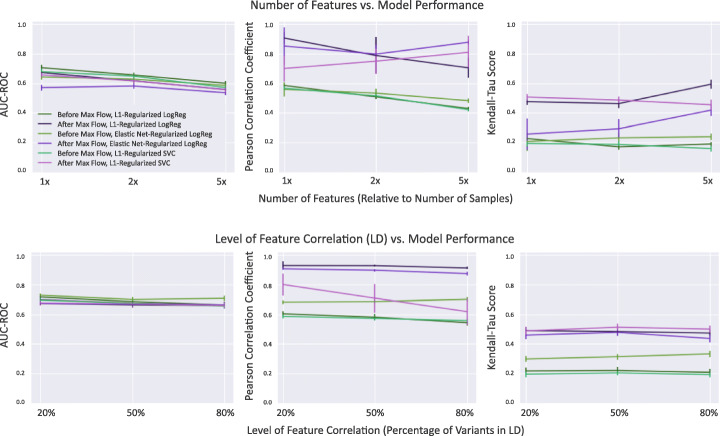
*Feature stability metrics on simulated data.* The graphs above show that the implementation of the maximum flow algorithm improves feature stability across various levels of feature correlation and dataset sizes. The maximum flow procedure also maintains prediction performance (AUC-ROC). Since the Jaccard index is trivially 1.0 after maximum flow, these values have been omitted from the figure

Specifically, we provide results for two types of simulations: (1) effect of dimensionality and (2) effect of feature correlation on performance of the maximum flow approach. For experiment (1), we test our model with three different dimensionalities (1000 features, 2000 features, and 5000 features) and set the level of feature correlation to be constant at 50%. Features are randomly divided into 23 groups of arbitrary sizes in order to represent chromosomes. With probability *p* = 0.5, each feature will be assigned a vector of values equivalent to that of another feature in the same chromosome group with Gaussian noise applied. For experiment (2), we test our model with three different levels of feature correlation (20, 50, and 80%). The number of features is held constant at 1000. AUC-ROC scores, Pearson correlation coefficients, Kendall-Tau scores, and Jaccard indices were computed for each model both before and after the implementation of maximum flow (Fig. 3).

Results show that the maximum flow approach leads to consistent improvements in model stability without compromising prediction performance. As shown in Fig. 3, the AUC-ROC of all three models remains fairly consistent after the implementation of the maximum flow algorithm, showing that the reduction in features does not negatively affect classification accuracy. In addition, Pearson correlation coefficients and Kendall-Tau scores show consistent improvements after the implementation of maximum flow, suggesting that our proposed method can improve feature stability under a variety of regularization types, feature correlation levels, dataset sizes, and models. Generally, the maximum flow approach appears to work best at higher dimensionalities, where there are at least 2–5 times as many features as samples, as well as moderate levels of feature correlation ranging between 20 and 50%. The maximum flow algorithm is sensitive to the level of regularization and type of model.

### Biological validation

We conducted a literature search to understand the effect of the 55 stable SRS variants on disease phenotype [20]. Since the impact of noncoding variants on disease manifestation is largely unknown, there are few functional annotations available for the noncoding genome. Many identified variants occur in undefined intergenic regions of the genome, with no known functional effects; 25 variants occur in intronic, upstream, or untranslated regions of protein-coding or noncoding genes. A variant located at position 127,832,148 on chromosome 10 is found in the intronic region of ADAM12, a gene that is known to be significantly expressed in placental tissue [21]. ADAM12 gene expression has also been linked to human intelligence through a genome-wide association study (GWAS) and the expressed protein serves as a maternal marker for Down’s syndrome; this evidence suggests an association with neural function [22, 23]. Similarly, a variant in chromosome 2 at position 104,690,808 is located in the intronic region of the noncoding RNA gene LINC01965, which GWASs have associated with Alzheimer’s Disease and ADHD; these disorders share clinical features with ASD and are consequently likely to possess similar underlying genetic pathways [24–26]. Additional annotations are listed in Additional file 1.

## Discussion

The recent revolution in low-cost sequencing technologies as well as the development of high-performance computational infrastructure have contributed to the discovery of numerous predictive variants associated with disease phenotypes. However, many standard machine learning methods for elucidating the relationship between genotypes and phenotypes are hindered by the presence of high-dimensional feature spaces, which can lead to model instability. The impact of our work is two-fold, contributing to advancements in machine learning model development as well as improvements in the genomic understanding of ASD and other complex multigenic human conditions.

We first develop an algorithm based on maximum flow, which utilizes the presence of linkage disequilibrium in order to perform dimensionality reduction. By using traditional feature elimination methods (like L_1_ regularization) and the maximum flow approach in succession, we were able to utilize biological knowledge to identify a core subset of stable, putative variants. To the best of our knowledge, such a method has never been used before for analysis of high-dimensional datasets. Harnessing information provided by linkage relationships between variants can allow for effective filtration of high dimensional feature spaces, enabling accurate identification of important genomic features.

Second, we utilize this method to perform a targeted investigation of the noncoding genome, examining the effects of variants in simple repeat sequences on the ASD phenotype. The contribution of noncoding variants to complex developmental disorders like ASD has been debated in recent years [27–29]. Our previous work suggested a correlation between variants in SRS regions and the ASD phenotype [19]. In this work, we extracted a list of 55 putative variants, which were demonstrated to be highly stable, as shown by a cross-validation analysis.

This study has some limitations. Variants in repetitive regions, especially those in unstable expansions, are difficult to call, which could potentially result in low quality reads. In addition, since the noncoding region is sparsely annotated, the majority of variants identified in this analysis have no known function, so additional biological verification is needed to validate these findings. Also, the greedy path search through the generated flow network possesses inherent randomness, with repeated runs and varied fold ordering resulting in slightly differing paths; however, our additional testing showed that this does not have a major effect on results. A path search performed on 120 different orderings of the five folds within the flow network resulted in a mean of 52.55 shared variants between pairs of orderings; similarly, 50 repeated runs of the algorithm resulted in a mean of 52.48 shared variants.

## Conclusion

In summary, we determined a set of 55 stable SRS variants potentially associated with the ASD phenotype. The methodology designed in this work allows for the creation of robust, interpretable, and scalable machine learning models that can effectively identify predictive variants from a high-dimensional feature space. Our results help pave the way towards biomarker-based diagnosis methods for ASD and other complex genetic conditions.

## Methods

We present an approach to identify genomic regions that remain stable despite slight perturbations to the underlying data. We begin by creating a regularized machine learning classifier to predict a disease phenotype from genomic regions; then, we implement an algorithm based on maximum flow to identify and extract stable features. We apply our technique to ASD and validate our findings.

### Measuring feature stability

We begin by constructing a binary feature matrix to encode the genome, with rows representing samples and columns corresponding to genomic regions. Genomic regions can encompass a variety of features, such as single nucleotide polymorphisms (SNPs), variants, or genes; in this analysis, we focus specifically on variants, which include both SNPs and short indels. In order to generate a binary representation, we assigned each cell a 1 if the sample expressed a homozygous alternate or heterozygous variant and a 0 if the base pairs at the site matched the reference sequence.

Then, we create a training set containing 80% of the samples in the dataset. A model with stable features will be resistant to slight perturbations in the underlying data, which increases confidence that top-ranked features are correlated with the phenotype [30, 31]. In a machine learning setting, cross-validation can be utilized as a method for perturbing the underlying data used to train a model and obtaining stability measurements [32]. In order to estimate feature stability, we perform k-fold cross validation across the training set with an *L*_1_-regularized logistic regression classifier. We select *k* = 5 in this work. For each of the five validation folds, we extracted the list of all variants with non-zero coefficient scores.

**Fig. 4:**
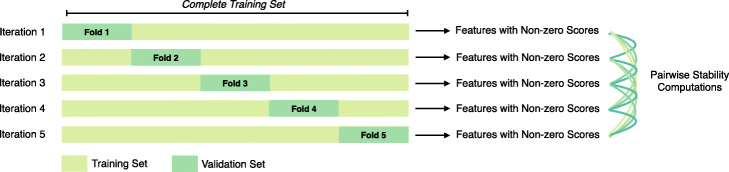
*Stability Measurement Methodology.* In order to characterize the stability of the model, we used an approach based on cross-validation, in which we measure model performance across five splits of the training set. We extract the list of predictive features (which are assigned non-zero coefficient scores) and perform pairwise comparisons in order to quantify similarity

Three metrics were used to characterize stability: (1) Pearson correlation coefficient, which measures the similarity between the coefficient scores assigned to each variant by the logistic regression model, (2) Kendall-Tau scores, which calculate the correlation between the ranked variant lists, and (3) Jaccard similarity index, which measures overlap between two sets of features [32, 33]. Given two lists of features A and B with associated coefficient scores w and v, the Pearson correlation coefficient is computed as $\left[{\sum}_i\left({w}_i-\overline{w}\right)\left({v}_i-\overline{v}\right)\right]/\sqrt{\sum_i{\left({w}_i-\overline{w}\right)}^2{\sum}_i{\left({v}_i-\overline{v}\right)}^2}$, with values ranging between − 1 (perfect negative correlation) and + 1 (perfect positive correlation). Kendall-Tau scores are computed as P-Q/(P + Q), where P represents the number of concordant pairs and Q represents the number of discordant pairs when the features are arranged in rank order, and range between − 1 and 1. The Jaccard index is computed as ∣A ∩ B ∣ / ∣ A ∪ B∣, with values ranging between 0 (no overlap) and 1 (identical). We computed these metrics for all ten pairwise comparisons of feature lists. This presents an initial metric of stability prior to implementation of our maximum flow formulation (Fig. 4).

### Maximum flow formulation

In this section, we discuss the maximum flow algorithm, which is implemented as a post-processing step to extract stable variants from the outputs of the regularized machine learning model created in the previous section. Biological datasets often present the characteristic of feature interdependence, which can enable informed dimensionality reduction based on domain knowledge [34]. In this work, we use the presence of LD to group correlated variants identified as predictive by the 5-fold cross validation performed in the previous section. Such an approach ensures that the model remains resistant to perturbations in training data and outputs interpretable results that can be utilized to measure the contribution of individual variants to disease phenotype. Features identified by this method are stable variants that are potentially associated with the manifestation of ASD.

LD is a genetic phenomenon that results in non-random correlation between a group of variants or alleles. A pair of variants is said to be in LD if the observed frequency of a particular haplotype in the genome is higher than the expected frequency [35]. LD is typically measured using the *R*^2^ metric, which ranges from 0 to 1, with larger values representing stronger association between variants [36]. It is important to note that measurements of LD in a sample population tend to be sensitive to the presence of rare variants, since limited data affects resulting scores.

We hypothesize that the presence of LD accounts for model instability; it is likely that the classifier identifies the same predictive regions in each fold yet extracts different variants, creating the appearance of instability as observed in the results from the previous section. We now discuss a novel formulation of the maximum flow algorithm, in which we utilize the presence of LD to identify regions that are consistently present across folds; this results in a set of stable variants that are likely to be correlated with the phenotype.

**Fig. 5:**
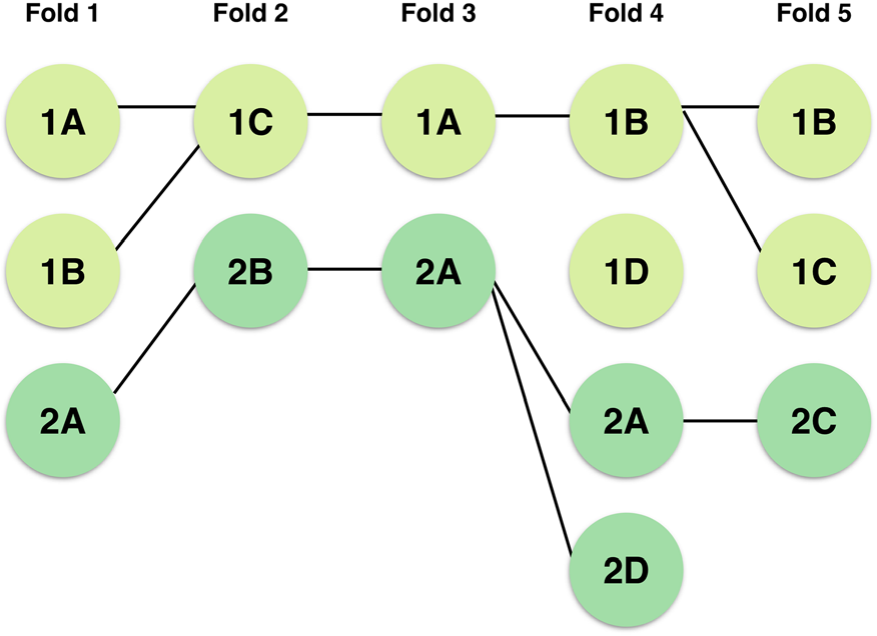
*Simplified depiction of graph model.* Consider a simplified representation of the dataset, consisting of variants 1A, 1B, 1C, and 1D (located on chromosome 1) as well as variants 2A, 2B, 2C, and 2D (located on chromosome 2). Assume that a feature matrix consisting of all eight variants served as input to an L_1_-regularized classifier, which performed five-fold cross validation and identified a set of top-ranked variants for each validation fold. In the depiction above, we see that the variants are clustered by fold, with an edge connecting a pair of nodes n_1_ and n_2_ if they are located in neighboring folds and have an R^2^ value greater than 0.8. Note that identical variants (such as variant 1B in folds 4 and 5) are trivially in LD

The 5-fold cross validation performed in the previous section results in five sets of variants with non-zero coefficient scores. We begin by creating a graph *G* to represent the presence of LD between pairs of these variants (4). Each node *n* in the graph is defined by a variant *v* as well as the fold in which it occurs *f*, which we represent as the tuple *n* = (*v,f*). Consider a pair of nodes *n*_1_ = (*v*_1_*, f*_1_) and *n*_2_ = (*v*_2_*, f*_2_); an edge is drawn between the pair if the following criteria are satisfied: (1) *n*_1_ and *n*_2_ are present in neighboring folds such that *f*_2_ = *f*_1_ + 1 and (2) *n*_1_ and *n*_2_ are in linkage disequilibrium as indicated by the *R*^2^ value between *v*_1_ and *v*_2_ exceeding 0.8 [36]. As a result of criterion (1), *G* is necessarily a 5-partite graph. Variants closer together in physical space are more likely to be in LD, so in this analysis, we compute LD within each chromosome; thus, an edge exists between nodes *n*_1_ and *n*_2_ if and only if *v*_1_ and *v*_2_ are located within the same chromosome. Consequently, G is composed of 23 disjoint subgraphs (Fig. 5).

We now utilize maximum flow to identify stable variants across folds [37]. We restructure *G* = (*N,E*) into a directed, acyclic flow network *L* = (*N,E*) such that it is amenable to the maximum flow formulation, a concept well studied in graph theory. To do so, we add a source node *s* and a sink node *t* to the graph. A directed edge is drawn between the source *s* and all nodes with a fold value of *f* = 1. Similarly, an edge is drawn between all nodes with a fold value of *f* = 5 and the sink *t*. To create the flow network *L*, we first define the capacity of an edge as the maximum amount of flow that can pass between nodes *n*_1_ and *n*_2_, defined as a real number *c*(*n*_1_*,n*_2_). We also define the flow between nodes *n*_1_ and *n*_2_ as a real number *l* limited by the capacity of the edge, such that *l*(*n*_1_*,n*_2_) ≤ *c*(*n*_1_*,n*_2_). With the exception of the source and sink nodes, the flow entering a node must equal the flow exiting the node. The total flow value of the graph can be computed as the flow that leaves the source node or enters the sink node, represented by the summation ${\sum}_{\left(s,n\right)\in E}l\left(s,n\right)$ = ${\sum}_{\left(n,t\right)\in E}l\left(n,t\right)$. Then, our goal is to maximize the total flow passing from the source to the sink node of a graph with respect to the criteria defined above.

**Fig. 6:**
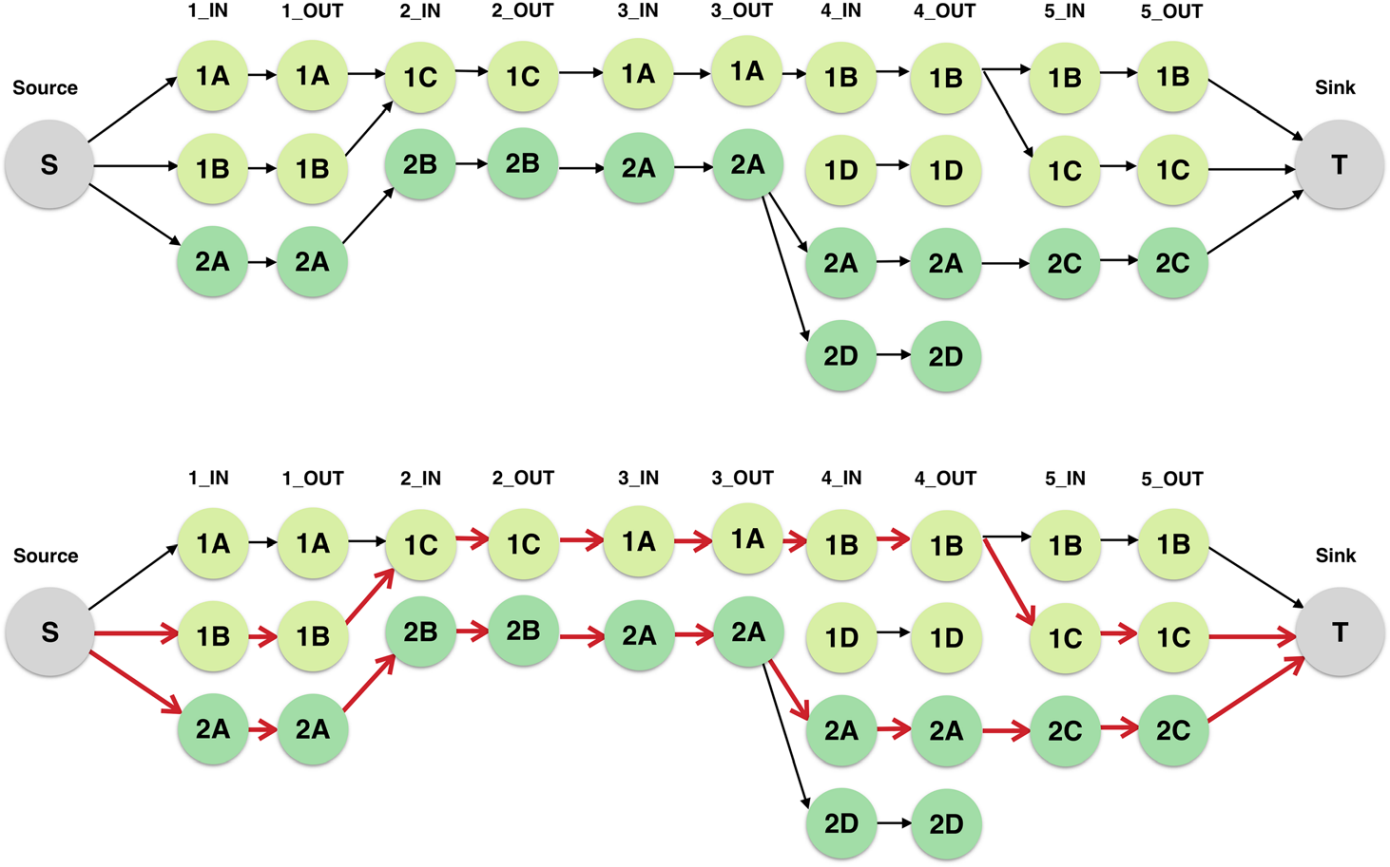
*Simplified depiction of maximum flow formulation.* (Upper) This is the flow network resulting from the graph created in Fig. 4. Consider a simplified representation of the dataset, consisting of variants 1A, 1B, 1C, and 1D (located on chromosome 1) as well as variants 2A, 2B, 2C, and 2D (located on chromosome 2). Source and sink nodes are added to the graph, and the variants in each fold are duplicated to constrain flow through the network. (Lower) A potential result of running the Ford-Fulkerson algorithm is shown here. The value of the maximum total flow through the graph is 2, as shown by the two paths (highlighted in red) that connect the source to the sink. We see from the graph that variants 1A, 1B, and 1C are part of a region of the genome that remains stable across multiple folds; this property can be noted for variants 2A, 2B, and 2C as well

In a k-partite graph, it is possible that the set of unique edges that define the maximum flow can include non-unique nodes; however, we seek to identify the maximum number of stable nodes across folds in order to identify regions in LD that are likely to influence the phenotype. Thus, we must constrain the flow network such that each node can only be used once within the flow. We do so by adding an additional customization to the network *L* = (*N,E*) to constrain flow. We split each node *n* = (*v,f*) into two nodes defined as *n*_*in*_ = (*v,f,in*) and *n*_*out*_ = (*v,f,out*), adding a single edge between *n*_*in*_ and *n*_*out*_. As a result of this modification, L doubles in size, becoming a 10-partite graph (excluding the source and sink nodes) (Fig. 6).

All edges in L are assigned a capacity of 1. Then, the flow through L is computed using the Ford-Fulkerson algorithm, a greedy path-search technique that identifies a maximal set of valid paths between the source and sink nodes [38]. The runtime of Ford-Fulkerson is *O* (*VE*^2^). The resulting maximum flow value defines the number of valid paths through the graph, and the nodes along each flow path from the source to sink represent a set of regions that remain stable across folds after accounting for the presence of LD.

### Data and preprocessing

We evaluated our methodology on 30x coverage whole genome sequence data collected from 2182 children with ASD and 379 control patients with progressive supranuclear palsy (PSP). The ASD population data was obtained from The Hartwell Foundation’s Autism Research and Technology Initiative (iHART), which has collected whole genome sequence data from 1006 multiplex families, with at least two children in each family presenting an ASD diagnosis [39]. Although case and control studies on ASD typically assign unaffected family members as controls, subclinical phenotypes of ASD that are often present in family members can suppress diagnostic signal. In order to overcome this issue, we selected to use a separate outgroup of patients with PSP as the control population. PSP is a neurodegenerative condition that has no etiological overlap with ASD and is generally not heritable. Only one gene is currently known to be linked with the condition. We provide further evidence to support the use of this control group in our prior work [19].

In order to limit batch effects due to sequencing methodologies, the PSP and ASD populations were both sequenced at the New York Genome Center with Illumina HiSeq X instruments. There is no overlap between the cohorts.

Previously, we showed that a regularized machine learning model trained on variants in Simple Repeat Sequences (SRS) was able to successfully differentiate ASD patients from the control group with high accuracy, suggesting that variation in SRS may be predictive of the ASD phenotype [19]. We now utilize the methods described in the previous sections to address the issue of feature instability and extract a robust set of SRS variants potentially correlated with the ASD phenotype.

SRS are segments of noncoding DNA that consist of repeating sequences of one to ten base pairs. These regions are highly susceptible to mutations, and unstable expansion of these regions has been linked to more than twenty neurodevelopmental and neurodegenerative conditions [40, 41]. We downloaded a list of chromosomal coordinates for all SRS from the UCSC Genome Browser, which identified 413,380 SRS regions in the human genome [42]. Variants likely to result from batch effects were removed using a genome-wide association test with batch (ASD and PSP) as the phenotype [19]. After preprocessing, variants present in both the ASD and PSP populations were identified, resulting in a final list of 232,193 SRS variants.

We create a binary feature matrix consisting of 2561 rows (corresponding to the 2182 ASD patients and 379 control patients) and 232,193 columns (corresponding to the SRS variants), which serves as input to the logistic regression classifier. To address class imbalance between the case and control populations, we adjusted classifier weights to be inversely proportional to class sizes. We then execute the maximum flow procedure as described in the previous sections and extract the list of variants determined to be stable across folds.

### Validation

We hypothesize that the presence of LD will cause the model to identify the same regions in each fold yet extract different variants. To evaluate this hypothesis, we determine whether the variants identified by the classifier across the five cross-validation folds are more stable than expected by random chance. We perform a bootstrap test, randomly selecting five sets of variants from the complete set of 232,193 SRS variants, maintaining sizes equivalent to those of the original folds. We construct a flow network using the procedure defined in the previous section and use this to compute maximum flow. We repeat this process 100 times. If our hypothesis is supported, we expect the random flow networks to be highly-disconnected due to low linkage between random variants. If our hypothesis is not supported, the random flow networks will result in connected graphs with flow values similar to those observed in our true network.

Next, we determine if a classifier trained on the variants identified by the maximum flow procedure is more stable than the original regularized machine learning model. To do so, we utilize the regions identified by the maximum flow algorithm to construct new feature matrices. Each flow path from the source to sink includes one to five unique variants in LD; this group of variants defines a region that appears to be stable across multiple validation folds. We construct a binary feature matrix with columns corresponding to the grouped regions. Each grouped network feature is assigned a 1 if at least one of those variants is present in the patient and a 0 otherwise. In order to demonstrate an improvement in feature stability, we perform five-fold cross validation across the training set with a logistic regression model trained on this reduced feature set. We recompute stability metrics (Pearson correlation coefficient, Kendall-Tau score, and Jaccard similarity index) for our reduced variant set and compare our results to the initial stability measurements that we obtained prior to implementation of the maximum flow algorithm.

In order to characterize the stability improvements afforded by the maximum flow approach, we conduct a series of experiments on simulated data. We use the make_classification function in the scikit-learn library to generate synthetic feature matrices for 2-class classification; the generated datasets consist of clusters of points distributed around vertices of a hypercube, with an approximately equal number of points assigned to each class [43]. This approach results in small effect sizes for individual features, which is reflective of standard genomic datasets. We divide features into 23 groups of arbitrary sizes in order to represent chromosomes, and we simulate linkage disequilibrium by introducing correlation between pairs of features located in the same chromosome. Then, we explore the effect of four parameters on the effectiveness of the maximum flow algorithm: level of feature correlation (percentage of variants in LD), dataset dimensionality (number of features), type of regularization (L_1_ or Elastic Net), and type of classifier (logistic regression or linear support vector classifier). For each experiment, we select parameters, compute baseline classifier performance and stability metrics, execute the maximum flow algorithm, and recompute performance and stability values with the smaller subset of features. Each experiment is repeated twenty times with random feature matrices.

Finally, we search for and characterize the enrichment of our highest ranked variants in biological roles associated with the autism phenotype.

## Supplementary Information

**Additional file 1.** SRS Variant Annotations. This extended table includes chromosomal coordinates and additional details for the 55 variants identified in this work.

## Data Availability

The data used in this paper is available to the public at ihart.org. Source code (implemented in Python) is available at https://github.com/maya124/MaximumFlow.

## Abbreviations

WGS: Whole Genome Sequence
ASD: Autism Spectrum Disorder
LD: Linkage Disequilibrium
SRS: Simple Repeat Sequence(s)
GWAS: Genome-Wide Association Study
