# Supplementary material for: A maximum flow-based network approach for identification of stable noncoding biomarkers associated with the multigenic neurological condition, autism
Source: BioData Min. 2021 May 3;14:28. doi: 10.1186/s13040-021-00262-x (PMC8091705; doi:10.1186/s13040-021-00262-x)
Supplement: Supplementary file 1 — Additional file 1. SRS Variant Annotations. This extended table includes chromosomal coordinates and additional details for the 55 variants identified in this work. [file 13040_2021_262_MOESM1_ESM.pdf]

SRS Variant Annotations						
Chromosome	Position (hg19)	Ref	Alt	RS ID	Location	Gene Annotations
1	83834087	C	T	rs116299817	LOC107985037, Intron Variant	
1	239337684	G	T	rs372460691	Intergenic	
1	4678570	CATATT	C	rs757584938	Intergenic	
1	193040980	ATATAAT	A	rs76411274	TROVE2, Intron Variant	TROVE2 is a protein-coding gene associated with autoimmune disorders (such as Lupus and Sjogren's syndrome)
2	145601462	A	C	rs1038363	TEX41, Intron Variant	TEX41 is a noncoding RNA gene
2	104690808	C	A	rs28397714	LINC01965, Intron Variant	LINC01965 is a long intergenic noncoding RNA gene. GWAS studies have linked this gene to Alzheimer's Disease and ADHD.
2	238120430	TGTGTAAGGTATGGGTGGTGTGTGTGATGTG TGTGTAATATGTATGTGGCTTGC	T	rs537095521	LOC105373953, 2KB Upstream Variant	LOC105373953 is a noncoding RNA gene
2	132530846	GAC	G	rs56020430	Intergenic	
3	22321137	TATA	T	rs199595457	ZNF385D, Intron Variant	ZNF385D (Zinc Finger Protein 385D) is a protein-coding gene associated with nucleic acid binding. A GWAS study has linked the gene to cognitive function measurements.
3	22321141	T	TC	rs62246559	ZNF385D, Intron Variant	ZNF385D (Zinc Finger Protein 385D) is a protein-coding gene associated with nucleic acid binding. A GWAS study has linked the gene to cognitive function measurements.
3	1750349	G	A	rs71611563	Intergenic	
4	74653975	GGAGGAGGATGAGAAA	G	rs139170855	Intergenic	
4	71278899	T	C	rs55954630	Intergenic	
4	133068140	TATGTCATATACATATACATATATATACAT	G	No RS ID Found	--	
5	25931621	A	C	rs202162174	Intergenic	
5	141988285	C	T	rs249925	FGF1, Intron Variant	FGF1 (Fibroblast Growth Factor 1) is a protein-coding gene involved in cell functions, such as growth, morphogenesis, and tissue repair
5	164898170	T	C	No RS ID Found	--	
6	104550663	A	T	rs3934526	Intergenic	
6	857907	C	T	rs4959466	Intergenic	
6	81641399	A	ATTTC	rs533475777	Intergenic	

6	47622814	GTATATATA	G	rs770474409	ADGRF2, 2KB Upstream Variant	ADGRF2 (Adhesion G Protein-Coupled Receptor F2) is a protein-coding gene associated with transmembrane signaling. A GWAS has linked this gene to Alzheimer's Disease
7	139772687	C	CAT	rs202152848	Intergenic	
7	124989542	A	G	rs62482064	LOC101928283, Intron Variant	LOC101928283 is a noncoding RNA gene. GWAS studies have linked this gene to antisaccade responses associated with schizophrenia and bipolar disorder.
8	3242171	C	T	rs13252543	CSMD1, Intron Variant	CSMD1 (CUB And Sushi Multiple Domains 1) is a protein-coding gene associated with smallpox and epilepsy.
8	69265858	T	C	rs7823422	C8orf34, Intron Variant	C8orf34 encodes a protein that regulates protein kinases.
9	70849504	C	G	rs1831501	Intergenic	
9	139082054	T	TCCTCCC	rs370337142	Intergenic	
10	127832148	T	C	rs11812621	ADAM12, Intron Variant	ADAM12 is a protein-coding gene associated with neurogenesis and human intelligence. It is enriched in placental tissue.
10	71218860	A	C	rs199809746	TSPAN15, Intron Variant	TSPAN15 encodes a protein that regulates cell development and other cellular functions.
10	89351921	GTA	G	rs71932345	Intergenic	
12	76708343	GA	G	rs145275229	Intergenic	
12	76708338	A	AG	rs149489976	Intergenic	
12	91500993	AGAGAAAG	A	rs768861215	LUM, Intron Variant	LUM (Lumican) is a protein-coding gene that is associated with corneal function and collagen fibril organization.
13	27904543	T	C	rs12868783	Intergenic	
15	22028258	T	C	rs201335039	Intergenic	
15	46738811	T	A	rs5003991	Intergenic	
16	87327716	C	G	rs8051585	LOC101928682, 2KB Upstream Variant	LOC101928682 is a noncoding RNA gene
16	9406952	ACTCAT	A	No RS ID Found	--	
16	8123686	ACG	A	rs201143864	LOC105371070, Intron Variant	LOC105371070 is a noncoding RNA gene
16	29282127	G	GTT	rs796214007	LOC105371159, 500B Downstream Variant	LOC105371159 is a noncoding RNA gene
17	14334500	A	T	rs11078247	Intergenic	

17	15671084	G	A	rs202032024	Intergenic	
18	2007182	TAG	T	rs761740541	Intergenic	
19	55332601	C	T	rs679057	KIR3DL1, Intron Variant	KIR3DL1 is a protein-coding gene that plays a role in immune response.
20	32077916	C	A	rs187449369	CBFA2T2, 5 Prime UTR Variant	
20	44386319	G	A	rs199803423	WFDC3, Intron Variant	WFDC3 is a protein-coding gene that inhibits peptidase and endopeptidase activity
20	44386321	A	ACATG	rs200207357	WFDC3, Intron Variant	WFDC3 is a protein-coding gene that inhibits peptidase and endopeptidase activity
20	10132717	T	A	rs368485066	SNAP25-AS1, Intron Variant	SNAP25-AS1 is a noncoding RNA gene. GWAS studies have linked this gene with cognitive impairment.
20	44386412	C	T	rs60864345	WFDC3, Intron Variant	WFDC3 is a protein-coding gene that inhibits peptidase and endopeptidase activity
20	53436814	AT	A	No RS ID Found	--	
X	92890041	TG	T	rs11358234	Intergenic	
X	22729779	TTA	T	rs61514845	PTCHD1-AS, Intron Variant	PTCHD1-AS (PTCHD1 Antisense RNA) is a noncoding RNA gene
X	30816398	T	C	rs62649735	Intergenic	
X	30807530	A	G	rs6631184	Intergenic	
X	118065919	C	G	rs6646296	Intergenic	
						Source: Gene functions from genecards.org and Entrez
